# Genetically Proxied Phosphodiesterase Type 5 (PDE5) Inhibition and Risk of Dementia: A Drug Target Mendelian Randomization Study

**DOI:** 10.1007/s12035-025-04732-9

**Published:** 2025-02-14

**Authors:** Stephen O. Brennan, Alexander C. Tinworth

**Affiliations:** 1 University of Galway, Galway, Ireland; 2 Mater Misericordiae University Hospital, Dublin, Ireland; 3 Clinical Trial Service Unit, Nuffield Department of Population Health, University of Oxford, Oxford, UK

**Keywords:** Dementia, Phosphodiesterase Type 5, Mendelian randomization

## Abstract

Phosphodiesterase−5 (PDE5) inhibitors have gained interest as a potential treatment for dementia. However, current evidence is limited to observational and pre-clinical studies. We conducted a drug-target Mendelian randomization (MR) analysis to investigate the on-target effects of pharmacological PDE5 inhibition on dementia subtypes and related phenotypes. We selected variants from around the *PDE5A* locus associated with diastolic and systolic blood pressure, as well as circulating PDE5A levels, to create three instruments for genetically proxied PDE5A inhibition. Using two-sample MR, we validated the instruments against erectile dysfunction and pulmonary arterial hypertension before assessing their associations with dementia subtypes, dementia-related proteins, and neuroimaging traits. After correcting for multiple comparisons, genetically proxied PDE5 inhibition, per one SD lower in diastolic blood pressure, was associated with higher odds of Alzheimer’s disease (OR 1.09, 95% CI 1.07–1.11) and Lewy body dementia (OR 1.32, 95% CI 1.23–1.41), but a trend towards lower odds of vascular dementia across all instruments. Genetically proxied PDE5 inhibition was associated with both beneficial and adverse effects on brain MRI traits. This included lower volumes of white matter hyperintensities (SD change − 0.035, 95% CI − 0.025, − 0.045), indicating potential benefits, but also reduced volumes of other structures, including the thalamus, suggesting potential adverse effects. PDE5 inhibition was associated with the concentrations of several proteins implicated in dementia pathophysiology. Our findings suggest that while PDE5 inhibition may be associated with a lower risk of vascular dementia, possibly by preventing white matter hyperintensities, it may increase risk of Alzheimer’s disease and Lewy body dementia, warranting further investigation before clinical trials.

**Supplementary Information:**

The online version contains supplementary material available at 10.1007/s12035-025-04732-9.

## Introduction

Dementia is a major contributor to global morbidity and has a profound impact on individuals and societies [1]. Alzheimer’s disease, vascular dementia, and Lewy body dementia have distinct clinical and pathological features but share pathophysiological pathways involving oxidative stress and mitochondrial dysfunction that lead to neuronal death and cognitive decline [2]. Phosphodiesterase type 5 (PDE5) inhibitors, initially developed to treat erectile dysfunction, were later repurposed for pulmonary arterial hypertension (PAH). PDE5 inhibitors prevent the degradation of cyclic guanosine monophosphate (cGMP) in vascular smooth muscle cells and result in nitric oxide-mediated vasodilation [3]. The prospect of repurposing PDE5 inhibitors for the treatment of neurodegenerative diseases, including Alzheimer’s disease, has recently garnered considerable interest [4].

Preclinical studies suggest that PDE5 inhibition may protect against Alzheimer’s disease, vascular dementia, and Lewy body dementia [5]. Several mechanisms have been implicated, including nitric oxide-driven synaptic strengthening, tau hyperphosphorylation, SIRT1/PGC1α signaling, and improved cerebral blood flow [4, 6, 7]. Recent retrospective cohort studies have reported a reduced risk of Alzheimer’s disease with the use of PDE5 inhibitors [8–14]. However, these protective effects may have been influenced by methodological biases inherent in observational research, including reverse causality and residual confounding [15].

In the absence of randomized controlled trials (RCTs), genetic studies can provide valuable insights into the on-target effects of PDE5 inhibitors. Mendelian randomization (MR) employs genetic variation that naturally and randomly segregates within populations to explore causal relationships between exposures and outcomes [16]. Drug-target MR uses genetic variants that predict the levels or activity of drug-target proteins, effectively acting as proxies for the exposure of interest. These genetic variants, which are unaffected by reverse causation and environmental confounders, can provide unique insights into the causal relationship between pharmacological agents and diseases [17]. Drug-target MR has previously been used to investigate the effects of PDE5 inhibitors on male fertility and overall well-being [18]. In this study, we aim to explore the potential benefit of PDE5 inhibition on dementia-related outcomes using a drug-target MR framework.

## Methods

### Study Design

In this study, we used MR to explore the causal association between genetically proxied PDE5 inhibition and 1) dementia subtypes, 2) brain imaging traits, and 3) neurodegeneration-associated proteins.

**Fig. 1:**
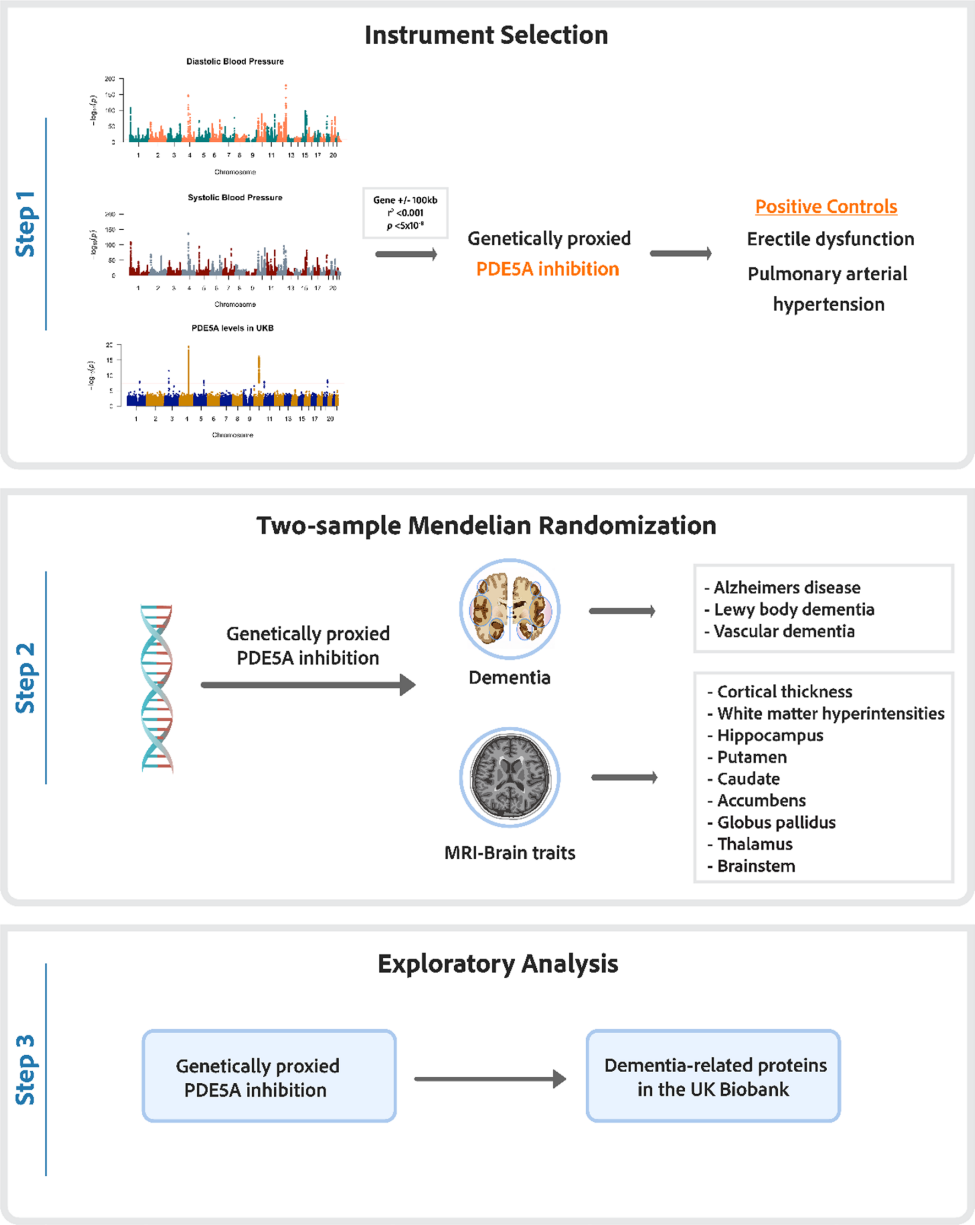
Overview of the study design. Stage 1 includes Manhattan plots for circulating levels of PDE5A in the UK Biobank, and blood pressure traits in the International Consortium for Blood Pressure (ICBP) and UK Biobank. The red line indicates the genome-wide significance threshold (5 × 10⁻⁸)

We first validated our genetic instruments as proxies for pharmacological PDE5 inhibition by evaluating their effects on established positive controls: blood pressure, circulating levels of PDE5A, erectile dysfunction, and PAH. Two-sample *cis*-MR was then used to elucidate the relationship between on-target PDE5 inhibition and the risk of Alzheimer’s disease, vascular dementia, and Lewy body dementia, along with magnetic resonance imaging (MRI) volume measurements of brain structures and circulating levels of proteins associated with neurodegenerative diseases. Subsequent analyses assessed the bidirectional effects between genetic liability to dementia and circulating levels of PDE5A. An overview of the study design is detailed in Fig. 1.

### Selection of Genetic Instruments

We utilized genetic effect estimates on diastolic blood pressure as our primary exposure of interest, a downstream trait known to be affected by PDE5A activity. To validate the robustness of our findings, we repeated analyses using genetic effect estimates on circulating concentrations of PDE5A and systolic blood pressure.

Summary statistics for systolic and diastolic blood pressure were obtained from Evangelou et al.’s genome-wide association meta-analysis involving 757,601 individuals of European ancestry from the International Consortium for Blood Pressure (ICBP) and UK Biobank (UKB) [19]. Summary statistics for Olink-measured circulating PDE5A levels were obtained from the UKB Pharma Proteomics Project, which included 35,571 individuals of European ancestry [20].

We identified common (minor allele frequency > 0.01) *cis*-genetic variants associated with blood pressure or circulating PDE5A concentration at genome-wide significance (*p* < 5 × 10^–8^) within a 100-kb flanking region of the *PDE5A* gene (hg19: chromosome 4: 120,415,550–120,550,146). For each instrument, variants were clumped using a linkage disequilibrium (LD) threshold of *r*^2^ < 0.001 and a distance threshold of 150 kb, using data from the 1000 Genomes Project European reference panel. This methodology selected two variants each associated with systolic and diastolic blood pressure (one of which was common between both instruments), and two variants associated with circulating PDE5A levels, as detailed in Supplementary Table 1.

Instrument strength was assessed through the calculation of an *F*-statistic and a mean *F*-statistic > 10 was considered strong. For bidirectional MR analyses, we created instruments for each dementia subtype by selecting genetic variants associated with each outcome at genome-wide significance from throughout the genome. We clumped these variants using an LD threshold of *r*^2^ < 0.001 and a distance threshold of 10,000 kb. The variants used to genetically proxy all three dementia subtypes are detailed in Supplementary Table 2.

### Outcome Data Sources

#### Disease Outcomes

Summary statistics for positive controls were obtained from Bovijn et al.’s 2018 genome-wide association study of 6175 self-reported or physician-diagnosed cases of erectile dysfunction and Rhodes et al.’s GWAS of 2210 cases of PAH [21, 22]. For Alzheimer’s disease, we used Wightman et al.’s GWAS (39,918 cases and 358,140 controls) with the exclusion of UKB and 23andMe participants due to the inclusion of proxy cases, sample overlap, and privacy restrictions [23]. Summary statistics for vascular dementia were sourced from FinnGen data release version 11 (3116 cases and 433,066 controls), and for Lewy body dementia, from Chia et al.’s GWAS of pathologically or clinically diagnosed cases (2591 cases and 4027 controls) [24, 25].

#### Imaging Traits and Proteins Associated with Neurodegenerative Diseases

For brain imaging traits, we used summary-level GWAS data for the MRI-measured volumes of brain structures, mean cortical thickness, and white matter hyperintensities (sample range 33,224–51,665) [26, 27].

A recent large-scale study identified 21 proteins associated with risk of Alzheimer’s disease and Parkinson’s disease, with Parkinson’s disease sharing similar pathophysiological features to Lewy body dementia [28, 29]. The potentially causal association between PDE5 inhibition and 19 of these Olink-measured proteins was assessed using summary-level GWAS data from the UK Biobank Pharma Proteomics Project [20]. Detailed information on all data sources used in this study is available in the Supplementary Methods.

### Statistical Analysis

#### Mendelian Randomization Analyses

Causal estimates from MR analyses rely on three key assumptions: (i) relevance, where the genetic instrument must be associated with the exposure; (ii) independence, where the instrument is not associated with any confounders of the exposure-outcome relationship; and (iii) exclusion restriction, where the instrument affects the outcome solely through its effect on the exposure. Random-effects inverse-variance weighted (IVW) models were used in primary MR analyses, after harmonising the alleles in the exposure and outcome datasets. Cochran’s *Q* statistic was used to assess for heterogeneity in effect estimates.

#### Sensitivity Analysis

To identify potential pleiotropic pathways that may violate the third MR assumption, we used the Common Metabolic Disease Knowledge Portal, a comprehensive aggregation of human genetic data and functional genomic information [30]. We searched each variant on the portal and identified potential pleiotropic pathways as traits significantly associated with the variant at a threshold of *p* < 1 × 10^–8^. The robustness of exposure-dementia outcome relationships was tested using two-step cis-MR, a method that involves the implementation of two MR analyses (one to estimate the confounder/mediator-outcome association and one to estimate the exposure-outcome association) [31].

#### Reporting and Packages

Results from MR analyses are reported as odds ratios (OR) for binary outcomes and as standard deviation (SD) differences for continuous outcomes per SD change in PDE5 levels or a decrease of one SD in blood pressure. Results from bidirectional MR analyses are reported as the effect of a one-unit increase in the log odds of genetic liability for each dementia subtype.

To control for multiple comparisons, we applied a sequential Bonferroni correction across all analyses. For dementia outcomes, statistical significance was defined as *p* < 0.0056, calculated by dividing the nominal alpha of 0.05 by the product of 3 outcomes and 3 instruments. For brain imaging phenotypes, we further adjusted the threshold to *p* < 0.00011 to account for the 17 additional outcomes across 3 instruments (0.0056/[17 × 3]). Lastly, for plasma protein concentration outcomes, significance was set at *p* < 1.91 × 10^–6^ to account for the 19 protein measurements analysed (0.00012/[19 × 3]). Positive control outcomes were not adjusted for multiple comparisons, as they were part of the instrument validation process. A proxy variant with an LD *r*^2^ > 0.90 was substituted when an instrumental variant was unavailable in the outcome summary statistics. All analyses were performed in R (v4.2.1; R Foundation for Statistical Computing) using the TwoSampleMR, MendelianRandomisation, and TwoStepCisMR packages.

### Standard Protocol Approvals, Registrations, and Patient Consents

All contributing studies obtained appropriate informed consent from participants and received ethical approval prior to commencement. This study was conducted in accordance with the guidelines for Strengthening the Reporting of Observational Studies in Epidemiology–Mendelian Randomization (STROBE-MR) (see Supplementary Material).

## Results

### Instrument Validation

Each of our instruments for genetically proxied PDE5 inhibition had a mean *F*-statistic > 30 and our diastolic blood pressure-weighted instrument (per SD lower in blood pressure) was strongly associated with higher circulating levels of PDE5A in UKB (SD change 0.67, 95% CI 0.63, 0.71, *p* < 0.001). Higher genetically predicted PDE5A levels were associated with lower diastolic blood pressure (SD change − 0.91, 95% CI − 0.90, − 0.93, *p* < 0.001). This paradoxical relationship, where higher PDE5A levels are associated with lower enzyme activity and a reduction in downstream traits, may be similar to mechanisms observed in other drug-target MR studies, such as those involving the IL-6 receptor [32].

### Positive Controls

**Fig. 2:**
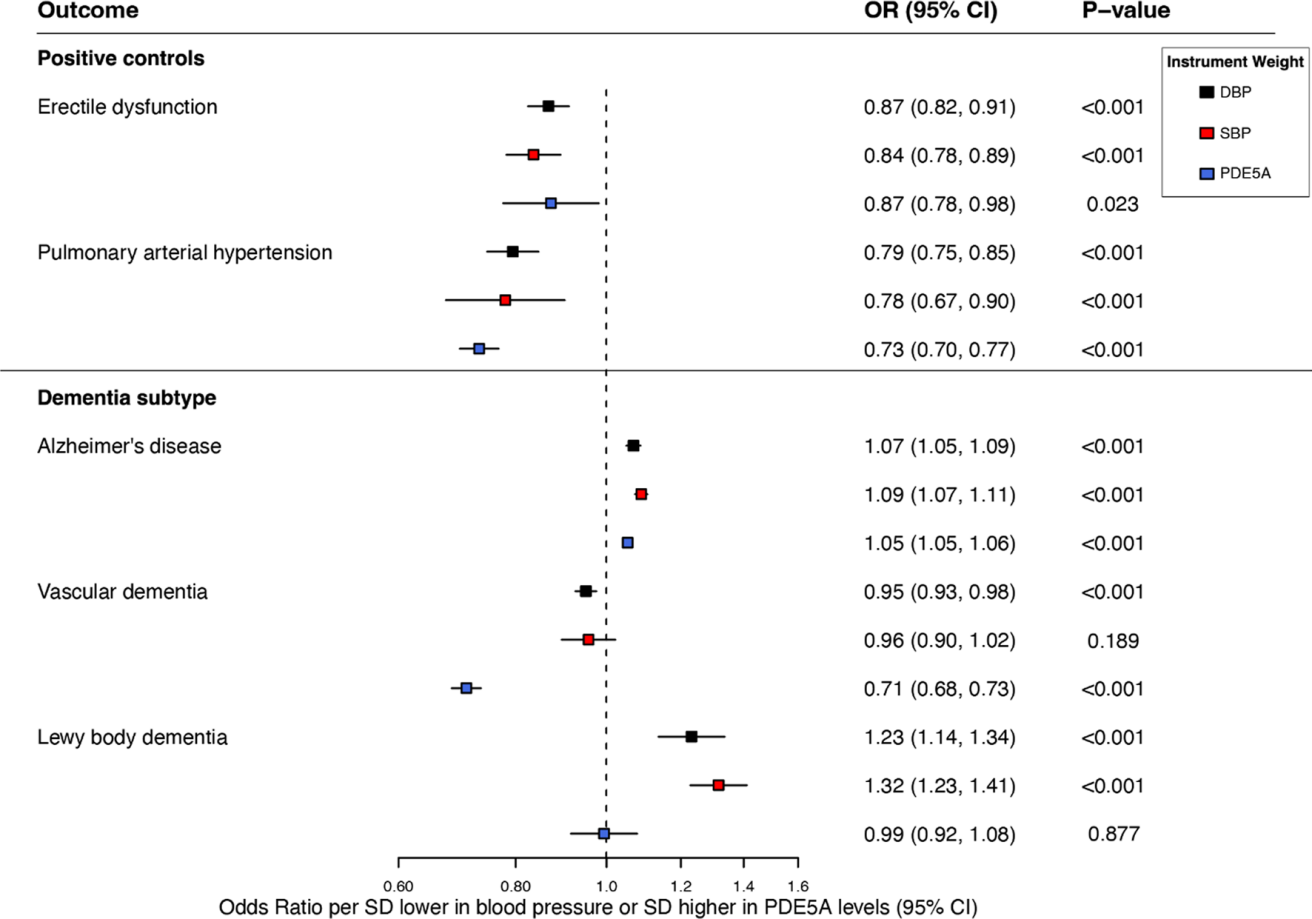
Association between genetically proxied PDE5 inhibition, positive controls, and dementia subtypes across genetic instruments. Results are derived from random-effects inverse-variance weighted Mendelian randomization analyses. OR indicates odds ratio; CI, confidence interval; SD, standard deviation

Genetically proxied PDE5 inhibition was associated with 16% lower odds of erectile dysfunction (OR 0.84, 95% CI 0.78–0.89, *p* < 0.001) and 22% lower odds of pulmonary arterial hypertension (OR 0.78, 95% CI 0.67–0.90, *p* < 0.001) per SD lower in diastolic blood pressure. Similar results were observed for our protein quantitative trait loci (pQTL) and systolic blood pressure-weighted instruments (Fig. 2).

### Dementia Subtypes

Genetically proxied PDE5 inhibition was associated with higher odds of Alzheimer’s disease (OR 1.09, 95% CI 1.07–1.11, *p* < 0.001) and Lewy body dementia (OR 1.32, 95% CI 1.23–1.41, *p* < 0.001) per SD lower in diastolic blood pressure (Fig. 2). Similar results were observed when using our pQTL and systolic blood pressure-weighted instruments for Alzheimer’s disease, but the protein level-weighted instrument was not associated with Lewy body dementia. Both our pQTL-weighted (OR 0.71, 95% CI 0.68–0.73, *p* < 0.001) and our systolic blood pressure-weighted instruments (OR 0.95, 95% CI 0.93–0.98, *p* < 0.001) were significantly associated with lower odds of vascular dementia. However, while directionally consistent, our diastolic blood pressure-weighted instrument displayed no significant association with vascular dementia (OR 0.96, 95% CI 0.90–1.02, *p* = 0.189). As indicated by Cochran’s *Q* statistic, no heterogeneity was observed across any of the instruments and their associations with dementia subtypes (*Q*: *p* = 0.52–0.89) (Supplementary Table 3).

#### Bidirectional Relationship Between Dementia Subtype and PDE5A Levels

In reverse MR analyses, genetic liability to Alzheimer’s disease was not significantly associated with circulating levels of PDE5A in UKB (*β* representing the effect of one unit increase in the log odds of genetic liability for Alzheimer’s disease 0.001, 95% CI − 0.017, 0.018, *p* = 0.95). Similarly, circulating levels of PDE5A were not affected by genetic liability to Lewy body dementia (*β* 0.004, 95% CI − 0.007, 0.015, *p* = 0.428) or vascular dementia (*β* 0.002, 95% CI –0.012, 0.016, *p* = 0.79). No significant heterogeneity was detected (*Q*: *p* = 0.70–0.87) (Supplementary Table 4).

#### Brain Imaging Phenotypes

**Fig. 3:**
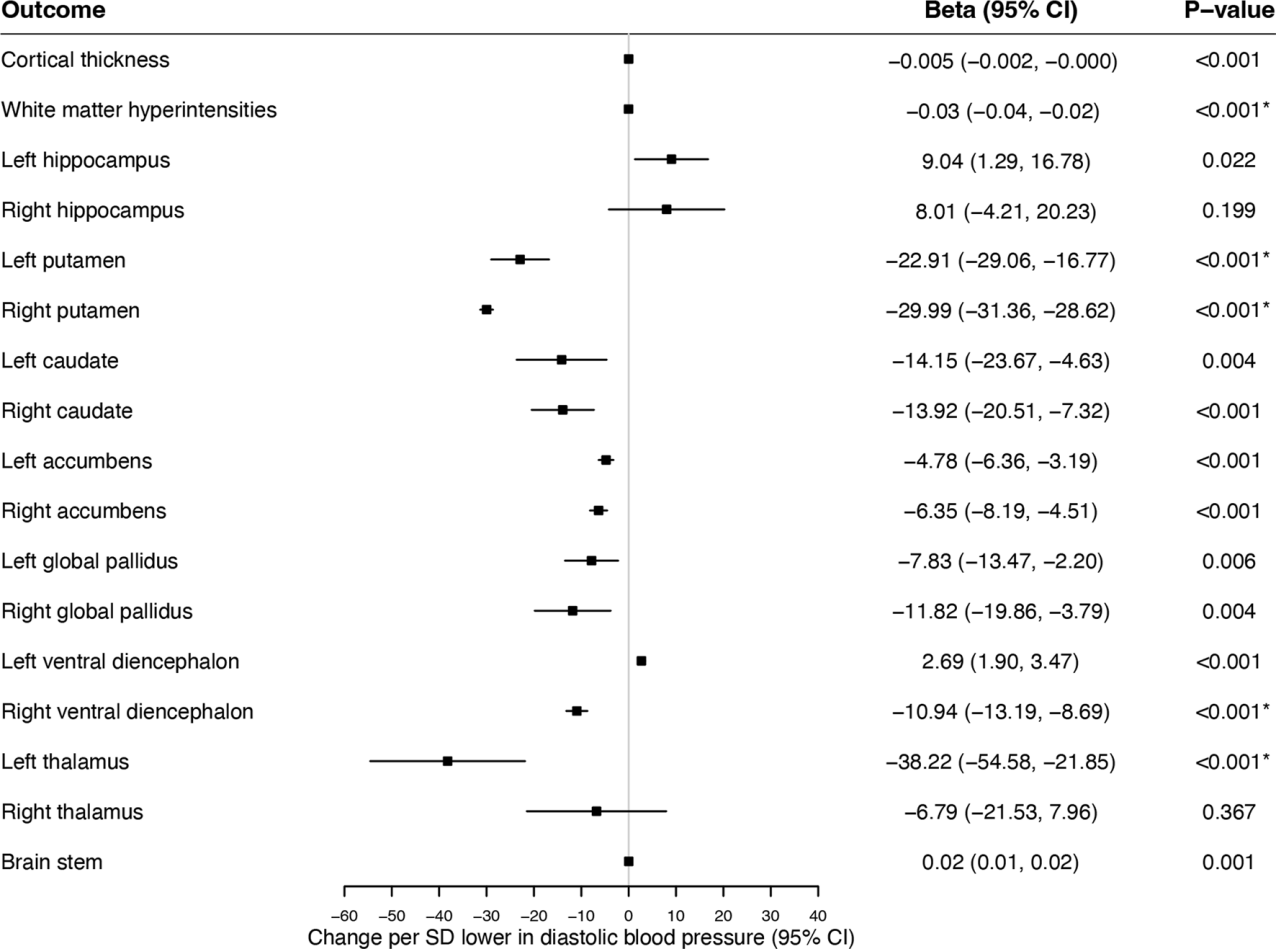
Association between genetically proxied PDE5 inhibition and magnetic resonance imaging brain traits. Results are derived from random-effects inverse-variance weighted Mendelian randomization analyses. OR indicates odds ratio; CI, confidence interval; SD, standard deviation. Outcomes marked with * indicates those that reached the sequential Bonferroni significance threshold (*p* < 0.00011)

Of the 16 brain imaging phenotypes analysed, genetically proxied PDE5 inhibition was associated with lower white matter hyperintensity volume (per SD change in diastolic blood pressure − 0.035, 95% CI − 0.025, − 0.045, *p* < 0.00012), left putamen volume (SD change − 22.915, 95% CI − 16.773, − 29.056, *p* < 0.00012), right putamen volume (SD change − 29.987, 95% CI − 28.617, − 31.357, *p* < 0.00012), right ventral diencephalon volume (SD change − 10.939, 95% CI − 8.690, − 13.187, *p* < 0.00012), and left thalamus volume (SD change − 38.218, 95% CI − 21.853, 54.583, *p* < 0.00012) (Fig. 3). These associations remained significant across both the systolic blood pressure and pQTL-weighted instruments (Supplementary Figs. 1–2). No significant heterogeneity was detected (*Q*: *p* = 0.54–1.00) and the full results across all three instruments can be viewed in Supplementary Table 3.

#### Neurodegenerative-Related Proteins

**Fig. 4:**
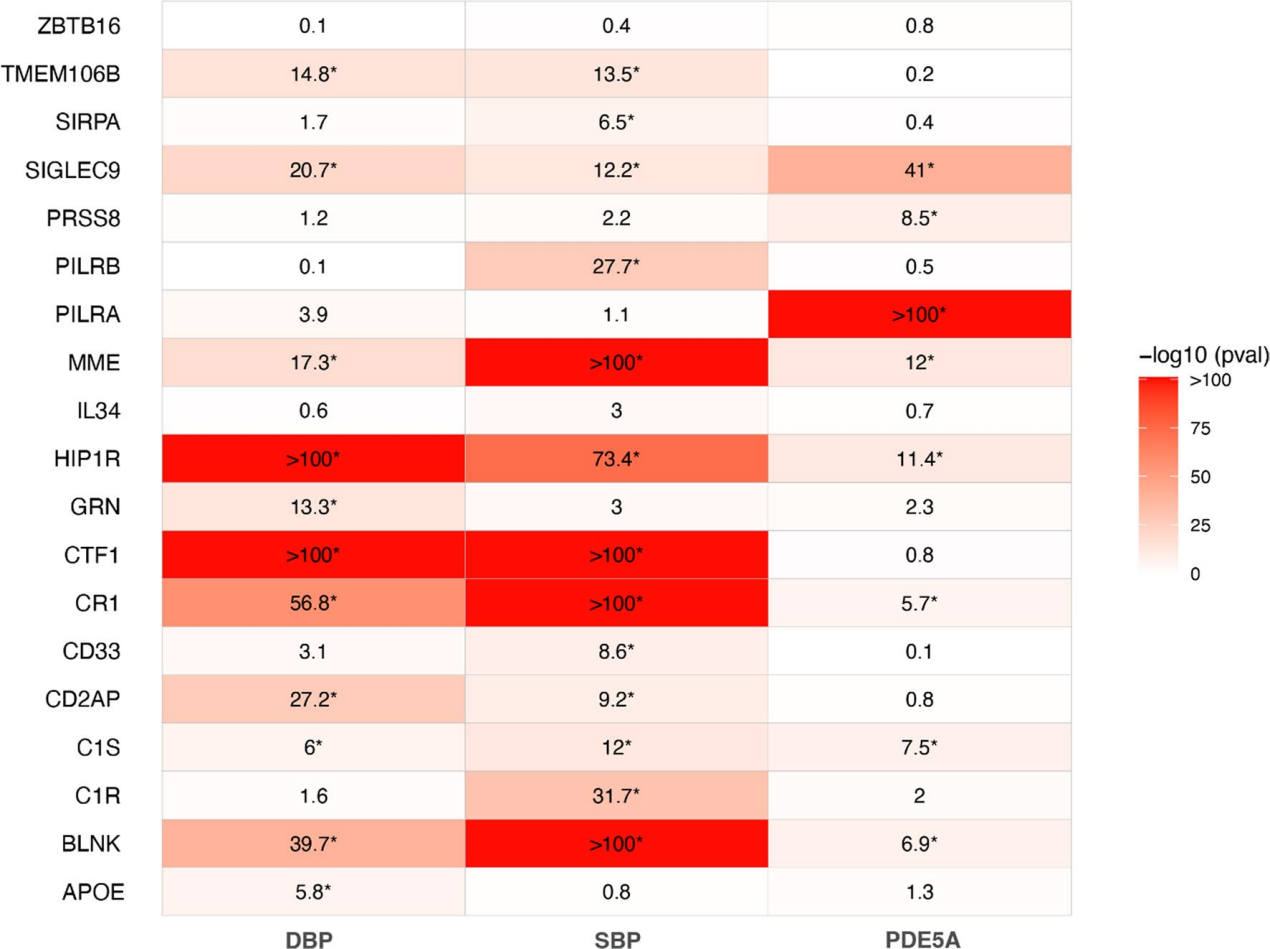
Heatmap displaying the association between genetically proxied PDE5 inhibition and circulating levels of neurodegenerative-related proteins in the UK Biobank. Results are derived from random-effects inverse-variance weighted Mendelian randomization analyses across instruments for PDE5A inhibition, Outcomes marked with * indicate those that reached the sequential Bonferroni significance threshold (*p* < 1.91 × 10^–6^**)**

Of the 19 proteins analyzed, genetically proxied PDE5 inhibition was significantly associated with lower plasma protein concentrations of CR1 (per SD change in diastolic blood pressure − 0.092, 95% CI − 0.081, − 0.104, *p* < 1.91 × 10^–6^), BLNK (SD change − 0.158, 95% CI − 0.135, − 0.182, *p* < 1.91 × 10^–6^), C1S (SD change − 0.041, 95% CI − 0.025, − 0.058, *p* < 1.91 × 10^–6^), SIGLEC9 (SD change − 0.047, 95% CI − 0.037, − 0.056, *p* < 1.91 × 10^–6^), HIP1R (SD change − 0.113, 95% CI − 0.108, − 0.118, *p* < 1.91 × 10^–6^), alongside higher concentrations of MME (SD change 0.040, 95% CI 0.031, 0.049, *p* < 1.91 × 10^–6^) (Fig. 4). These associations remained significant for both the systolic blood pressure- and pQTL-weighted instruments. However, for CR1, the pQTL-weighted instrument showed an inverse effect compared to the blood pressure-weighted instruments. No significant heterogeneity was detected (*Q*: *p* = 0.66–0.99). Full results can be viewed in Supplementary Table 3.

#### Pleiotropic Effects

Using the Common Metabolic Diseases Knowledge Portal, we identified seven traits associated (*p* < 5 × 10^–8^) with at least one of the variants included in our diastolic blood pressure-weighted genetic instrument (Supplementary Table 5). In two-step cis-MR, none of these traits significantly influenced the relationship between genetically proxied PDE5 inhibition and either Alzheimer’s disease or Lewy body dementia (Supplementary Table 6). No heterogeneity was observed across all MR analyses, as indicated by Cochran’s *Q* statistic (Supplementary Tables 3–6).

## Discussion

This study utilized large-scale genomic data from clinical, proteomic, and imaging phenotypes to provide novel insights into the relationship between PDE5 inhibition and dementia. Genetically proxied PDE5 inhibition was associated with a reduction in MRI-measured white matter hyperintensities and showed a potential protective effect on the risk of vascular dementia. However, it was also associated with several neurodegenerative-related proteins, a reduction in the volume of several components of the forebrain, and an increased risk of Alzheimer’s disease and Lewy body dementia.

Evidence from experimental models and observational studies implicates PDE5 in the pathogenesis of age-associated neurodegenerative disorders. PDE5 is expressed in brain neurons and vascular myocytes within subcortical white matter, where it degrades cGMP, regulates vasoconstrictor tone and maintains vascular homeostasis [4]. PDE5 and cGMP are involved in nitric oxide-dependent neurogenesis, synaptic plasticity, and mitochondrial function [4]. In vivo, PDE5 inhibition promotes neurite growth and reduces phospho-tau expression in neuron models derived from induced pluripotent stem cells from Alzheimer’s disease patients [8]. In rodent models of dementia, PDE5 inhibition enhances memory performance and reduces hyperphosphorylated tau levels [33]. Additionally, cGMP has also been implicated in the expression of enzymes involved in amyloid-β production [34].

Some, but not all, retrospective observational studies have reported an association between the prescription of PDE5 inhibitors and a lower risk of dementia in patients with erectile dysfunction and PAH [8–14]. A meta-analysis of six observational studies found a protective association between PDE5 inhibitor use and reduced risk of Alzheimer’s disease. However, these results demonstrated significant heterogeneity (*I*^2^ = 99%) and the quality of evidence was rated as very low [35]. The prolonged prodromal phase that precedes a formal diagnosis of dementia poses significant challenges for observational studies assessing the impact of exposures on the risk of dementia [36]. This period, characterized by subtle progressive neurodegenerative changes, can lead to reverse causality, in which the early stages of dementia influence the likelihood or level of an exposure [37]. Failure to account for this prodromal stage can bias associations. This phenomenon is observed with sedative-hypnotic medications, where an apparent association between benzodiazepines and dementia did not persist after excluding studies that failed to account for reverse causation and confounding by indication [38]. Similarly, studies on PDE5 inhibitors that excluded the early years of follow-up or used a true active comparator design did not find a protective association between PDE5 inhibitors and dementia [13, 14].

Clinical studies have yet to demonstrate a significant beneficial association between PDE5 inhibitors and risk of dementia. Several small trials have assessed surrogate markers of cerebral blood flow in individuals with symptomatic small vessel disease, a group at high risk for vascular dementia. The PASTIS RCT observed no significant difference in cerebral blood flow between tadalafil and placebo in older individuals with symptomatic small vessel disease [39]. Similarly, Wang et al. found that PDE5 inhibition was associated with decreased verbal fluency and memory and had negative effects on cerebral hemodynamics [40]. More recently, in the OxHarp study, sildenafil did not reduce cerebral pulsatility but increased cerebrovascular reactivity and perfusion [41]. A recent MR study suggests that PDE5 inhibition is associated with protective effects on several cardiometabolic phenotypes, including coronary artery disease, ischaemic stroke, and chronic kidney disease [42]. Our findings expand on this evidence and support the exploration of PDE5 inhibitors as a potential therapeutic strategy to reduce white matter hyperintensities and lower risk of vascular dementia.

In contrast to preclinical and some observational studies, our findings suggest that PDE5A inhibition may be associated with an increased risk of Alzheimer’s disease. Our data is supported by evidence from a recent proteomic study that showed higher circulating PDE5A levels contributed to a weighted composite score, which predicted incident Alzheimer’s disease and memory deterioration in the Framingham Heart Study Offspring cohort [43]. We demonstrate that PDE5 inhibition is associated with atrophy in several subcortical structures, including the thalamus and putamen, regions previously linked with Alzheimer’s disease and impaired global cognitive performance [44]. However, we also found an increase in hippocampal volume with several of our instruments for PDE5 inhibition, which may suggest a higher risk of the hippocampal-sparing subtype of Alzheimer’s disease. Alternatively, this observation may align with preclinical studies suggesting that PDE5 inhibition enhances memory pathways which are focused within the hippocampus [6]. Despite this, chronic PDE5 inhibition may contribute to subcortical degeneration and potentially lead to a broader neurodegenerative process. This dual effect may explain why some studies have shown benefits with PDE5 inhibition for certain short-term cognitive functions but raises concerns about long-term neurodegenerative outcomes.

Our results suggest that PDE5 inhibition is causally associated with several proteins implicated in the pathophysiology of neurodegenerative diseases, suggesting potential mechanisms that could explain its deleterious effects. Complement receptor type 1 (CR1), complement component 1 s (C1s), sialic acid-binding Ig-like lectin 9 (SIGLEC9), and membrane metalloendopeptidase (MME) are important proteins involved in neuroinflammation and the clearance and degradation of amyloid beta. B-cell linker (BLNK) is essential for normal B-cell development, and depletion of B cells may reverse the progression of Alzheimer’s disease [45, 46].

SIGLEC9 and C1s play important roles in the immune response against bacterial pathogens. Microglia express multiple proteins of the SIGLEC family, several of which have been associated with neurodegenerative diseases [47]. The C1 complex (comprising C1q, C1r, and C1s) initiates the classical pathway of the complement system and is implicated in synapse pruning and loss [48]. The *C1s* gene was a significant hit in a recent GWAS of Alzheimer’s disease [49], and reduced levels of C1s have been observed in the cerebrospinal fluid of Alzheimer’s disease patients [50]. Although direct evidence of an association between PDE5 or cGMP signaling and SIGLEC9 or C1s in the central nervous system is limited, cGMP has a well-established relationship with microglial activity [51]. This suggests that SIGLEC9, C1s, and cGMP may interact within overlapping regulatory networks involved in the control of neuroinflammation.

Neprilysin, encoded by the MME gene, is a membrane metallopeptidase responsible for degrading small peptides, including amyloid-beta. Neprilysin activity declines with age, contributing to amyloid-beta accumulation [52], and post-mortem brain samples from Alzheimer’s disease patients show reduced NEP expression and activity [53]. While genetic variations in *MME* have been linked to increased Alzheimer’s disease risk, these effects may not be mediated by changes in NEP expression or activity [54]. In our study, genetically predicted PDE5A inhibition was associated with higher MME levels and an increased risk of Alzheimer’s disease. PDE5A and neprilysin exert opposing effects on cGMP signaling: PDE5A degrades cGMP, while neprilysin indirectly modulates cGMP levels through the degradation of peptides influencing its production [55]. Elevated cGMP levels from PDE5A inhibition may upregulate MME expression as a compensatory response to altered signaling dynamics. However, higher predicted MME expression may not correspond to increased neprilysin activity, as factors including post-translational modifications, enzyme inhibition, or age-related declines in neprilysin activity could limit amyloid-beta degradation. Similarly, we found that higher PDE5A levels correlate with lower enzyme activity in positive controls analyses, suggesting that increased expression does not necessarily translate into functional activity, possibly due to regulatory mechanisms or feedback inhibition.

While novel treatments such as Lecanemab for early Alzheimer’s disease have shown some promise [56], there remains a lack of effective therapeutic options for small vessel disease and vascular dementia. For PDE5 inhibitors, the potential cardiovascular and cerebrovascular benefits could outweigh the possible risk of Alzheimer’s disease and Lewy body dementia observed in this study. We found that the effect estimates for the association between PDE5 inhibition and disease were higher for Lewy body dementia than for Alzheimer’s disease. This finding, along with the protective relationship between PDE5 inhibition and white matter hyperintensities, suggests that PDE5 inhibition may increase the risk of dementia through pathways independent of endothelial function. Because drug-target MR proxies lifetime exposure to a pharmacological intervention, the results of this study should not be interpreted as evidence that the use of PDE5 inhibitors for current licensed indications is associated with Alzheimer’s disease. Instead, our findings contradict evidence from preclinical and observational studies that suggested PDE5 inhibition could be a suitable therapeutic target for Alzheimer’s disease.

### Strengths

The primary strength of this study lies in the development and implementation of a genetic instrument that reliably proxies PDE5 inhibition. Unlike observational studies, this approach is robust to confounding factors and reverse causation. The validity of our results is further supported by successfully replicating the known pharmacological effects of PDE5 inhibitors on erectile dysfunction and PAH.

Case ascertainment of each dementia subtype was based on clinically diagnosed or pathologically confirmed cases, reducing the potential bias from including proxy dementia cases based on family history without confirmed clinical disease. The consistency of results across three genetic instruments and two-step cis-MR, which adjusts for potential pleiotropy, along with the triangulation of evidence from neuroimaging data and dementia-implicated proteins, strengthens confidence in our primary findings. Finally, the lack of bidirectional evidence, in which dementia is causally linked with PDE5A protein levels, further suggests robustness against reverse causality.

### Limitations

Several limitations should be considered when interpreting these results. Data was not available on the proportion of cases clinically diagnosed through imaging and cerebrospinal fluid biomarkers in large genetic consortia which raises potential concerns about case misclassification. Effect sizes derived from drug-target MR studies reflect lifelong perturbation of the drug target, rather than the short-term treatment duration typically seen in clinical practice, potentially leading to significant differences in the magnitude of benefit. Similarly, there may be temporal interactions between PDE5 inhibitors and neurocognitive disorders that do not become clinically relevant until the condition has reached a certain level of pathophysiology. Additionally, the *PDE5A* gene can encode several subtypes of PDE5, including PDE5A3, which is specifically expressed in vascular smooth muscle cells [57]. However, to date, assay-based protein plasma technologies are unable to differentially capture these isotypes, and the independent effects could not be explored in this study. Finally, summary statistics for dementia are predominantly available for individuals of European ancestry, limiting our ability to assess the effect of genetically proxied PDE5 inhibition across ancestries.

## Conclusion

This drug-target MR study has explored the effect of genetically proxied PDE5 inhibition on the risk of dementia and neurocognitive phenotypes. Findings indicate that PDE5 inhibition may be causally associated with a lower risk of vascular dementia but a higher risk of Alzheimer’s disease and Lewy body dementia. Analysis of neuroimaging traits and relevant proteins suggest both protective and harmful effects of sustained PDE5 inhibition. Further studies exploring these pathways are warranted prior to clinical trials of pharmacological PDE5 inhibition in the treatment and prevention of dementia.

## Supplementary Information

Below is the link to the electronic supplementary material.

Supplementary file1 (DOCX 301 KB)

Supplementary file2 (XLSX 32 KB)

## Data Availability

This study utilises publicly available, summary-level GWAS data that can be accessed through the GWAS Catalogue (https://www.ebi.ac.uk/gwas/home) or specific cohort portals, including FinnGen (https://www.finngen.fi/en/access_results), the Social Science Genetic Association Consortium (https://www.thessgac.org/), and ENIGMA (https://enigma.ini.usc.edu/).
