# Supplementary material for: Genetically Proxied Phosphodiesterase Type 5 (PDE5) Inhibition and Risk of Dementia: A Drug Target Mendelian Randomization Study
Source: Mol Neurobiol. 2025 Feb 14;62(6):7864–74. doi: 10.1007/s12035-025-04732-9 (PMC12078358; doi:10.1007/s12035-025-04732-9)
Supplement: Supplementary file 1 — Supplementary file1 (DOCX 301 KB) [file 12035_2025_4732_MOESM1_ESM.docx]

**Supplemental online content**

**Supplementary Methods**

**Study design**

Mendelian randomisation (MR) uses genetic variants associated with an exposure of interest, commonly obtained from genome-wide association studies (GWAS), to act as an instrumental variable when exploring causal relationships between that exposure and an outcome. Due to Mendel’s laws of segregation and independent assortment, genetic variants are randomly distributed throughout a population, and therefore mimic the randomisation seen in randomised controlled trials (RCT) [1]. Additionally, due to being assigned at conception, these instrumental variables do not suffer from reverse causation, a common bias seen in observational studies.

Estimates from MR analyses can be considered causal if three assumptions are met: (i) relevance, where the genetic instrument is associated with the exposure of interest, (ii) exchangeability, where the instrument is not associated with confounders of the exposure-outcome relationship, and (iii) the exclusion restriction assumption, where the instrument does not affect the outcome through a pathway separate to that of the exposure. The relevance assumption is often satisfied through generation of F statistics, where an F statistic above 10 is considered sufficient to avoid weak instrument bias, but the other two assumptions can only be partially addressed [2]. MR is continually updated with new methodologies that can mitigate potential violations of the three assumptions, with careful consideration required for their applicability depending on the research question.

Recently, the scope of MR (Mendelian Randomization) has broadened to include the investigation of drug targets. Most existing drugs target specific proteins and the assessment of genetic variants from around the gene loci of these proteins can be used to assess the effect of perturbing the drug target protein on outcomes of interest. Genetic variants within or near the locus of the gene encoding the drug target protein of interest are known as cis-variants, and these can be used in MR to investigate current or novel drug targets [3]. Drug target MR is still subject to the same assumptions as conventional MR, with additional focus on validation steps that ensure the genetic instrument captures the effect of its pharmacological intervention, often by checking for associations with positive control outcomes [4]. Another important consideration is that the investigated perturbation is measured over an individual’s lifetime, compared to an RCT where the intervention is over a shorter period [5].

**Data sources**

*Diastolic and systolic blood pressure*

Mean diastolic and systolic blood pressure was derived from two measurements in UK Biobank (UKB) (automated or manual) when available or for a small subset through a single measurement (N=413). Following quality control, 458,577 individuals from UKB were included, and blood pressure medications were adjusted for where appropriate. For the GWAS, association analyses were performed using a linear mixed model that adjusted for age, age^2^, body mass index, and the optional inclusion of covariates that controlled for population stratification. For the International Consortium for Blood Pressure (ICBP) GWAS, post-quality control data from 54 studies comprising 150,134 individuals was combined with 23 independent studies of 148,890 individuals in total resulting in a dataset of 299,024 individuals of European ancestry. Summary data from the UKB and ICBP were combined using a fixed-effects inverse variance weighted meta-analysis [6].

*Plasma PDE5A protein levels*

Summary statistics for plasma PDE5A protein levels were obtained from the UKB Pharma Proteomics Project (UKB-PPP) GWAS of 54,219 UK Biobank participants on 2,923 plasma protein concentrations measured using the antibody-based Olink Explore 3072 panel. Of this cohort, 46,595 participants were randomly selected, 6,376 were selected by the UKB-PPP consortium members, and 1,268 individuals who participated in the COVID-19 repeat imaging study [7]. Summary statistics used in this study were of European ancestry individuals used in the discovery protein quantitative trait loci (pQTL) discovery cohort (n = 34,557).

*Erectile dysfunction*

Summary statistics for erectile dysfunction were obtained from Bovijn et al.’s GWAS of 6,175 cases and 218,630 controls. The contributory studies were UKB, the Estonian Genome Center of the University of Tartu (EGCUT), and the Partners Healthcare Biobank (PHB). Erectile dysfunction was classed as self-reported or clinically reported using ICD10 codes N48.4 and F52.2. Cases also included individuals on medications such as sildenafil, tadalafil, vardenafil, or surgical history of erectile dysfunction intervention [8].

*Pulmonary arterial hypertension*

Pulmonary arterial hypertension was defined across the four included studies through haemodynamic criteria according to international guidelines, namely, an increase in mean pulmonary arterial pressure by ≥25 at rest through right heart catheter assessment [9]. Individuals were included if they were unrelated and cases resulting from autoimmune disease were excluded. SNP associations were assessed through logistic regression adjusting for sex, read length chemistry, and principle components (number depended on each individual study). Findings were cross-validated across all four studies using the inverse variance-weighted fixed effects meta-analysis approach [10].

*Dementia subtypes*

Alzheimer’s disease cases were obtained from Wightman et al.’s GWAS of late-onset AD from thirteen cohort studies. However, for the purposes of this study, two datasets (UKB and 23andMe) were excluded due to inclusion of proxy cases, sample overlap, and privacy restrictions. Case definition varied slightly between the datasets but were either registered to ICD10 codes F00 and G30, defined from medical assessment, or neurological evaluation, and controls were considered healthy with no prior diagnosis of Alzheimer’s disease [11]. For vascular dementia, cases in FinnGen (a cohort study with over 300,000 genotyped individuals of Finnish ancestry living in Finland) were recorded as ICD-10 F01 [12], and Lewy body dementia was defined by Chia et al. as clinically probable (802 cases) or through autopsy confirmation (1,789 cases). Controls were selected based on a lack of evidence of cognitive decline or neurological deficits on neurological examination [13].

*Neuroimaging traits*

Summary statistics for average cortical thickness were obtained from Grasby et al.’s genome-wide association meta-analysis of brain magnetic resonance imaging (MRI) data from 33,992 participants across 50 cohorts [14]. Summary statistics for the volume of the brainstem and white matter hyperintensities were obtained from Smith et al.'s GWAS, which included 33,224 UKB participants who underwent multimodal brain imaging [15]. White matter hyperintensities was assessed using T2-weighted FLAIR structural imaging, while brainstem volume was generated by parcellation of the white surface using Desikan-Killiany parcellation, a gyral-based atlas used to segment brain tissue into its various subcortical structures [16].

The additional brain volume summary statistics were obtained from a GWAS of 36,778 UK Biobank participants. Cortical surfaces modelling was performed using FreeSurfer, and 33 volumes were extracted based on Desikan-Killiany surface templates [17].

*Dementia implicated proteins*

Proteins implicated in Alzheimer’s disease and Parkinson’s disease, which shares similar pathophysiological features to Lewy body dementia [18], were identified from a pQTL-based Mendelian randomisation and colocalization study of proteins involved in several neurodegenerative studies [19]. Proteins were selected for our study if they passed the author’s false discovery rate correction for multiple testing and Colocalization posterior probability threshold of >0.80 for evidence of a shared causal variant. Of the 21 eligible proteins, 19 were measured using the Olink platform in the UKB-PPP and were included in the analysis.

*Traits identified for two-step cis-Mendelian randomisation*

All summary statistics used for the two-step cis-MR portion of the analyses were obtained from the Integrative Epidemiology Unit (IEU) OpenGWAS project in individuals of European ancestry [20]. Summary statistics for plateletcrit (n = 408,112, IEU OpenGWAS ID: ebi-a-GCST90002400) were obtained from a GWAS of the UKB and INTERVAL studies [21]. Coronary artery disease summary statistics (cases = 122,733, controls = 424,528, IEU OpenGWAS ID: ebi-a-GCST005195) was obtained from a GWAS of UKB and CARDIoGRAMplusC4D [22]. Lymphocyte (n = 524,923, Open GWAS ID: ebi-a-GCST90002316) and monocyte count (n = 521,594, OpenGWAS ID: ebi-a-GCST90002340) summary statistics were obtained from a GWAS of several European cohorts included UKB, INTERVAL, and the Million Veterans Programme (MVP) [23]. White blood cell count (n = 563,946, IEU OpenGWAS ID: ieu-b-30) and LDL cholesterol (n = 440,545, OpenGWAS ID: ieu-b-110) summary statistics were obtained from many consortia that were manually curated by the IEU for use in the MR-Base platform [24]. Platelet count (n = 350,474, IEU OpenGWAS ID: ukb-d-30080_irnt) summary statistics were extracted from GWAS conducted on UKB [25].

*Sensitivity analysis*

Heterogeneity between variants was assessed through Cochran’s Q statistic which follows an X2 distribution with degrees of freedom defined as n number of SNPs minus 1. A P-value <0.05 indicates a violation of the MR assumptions across some or all of the genetic variants [26]. Finally, traits identified by SNP lookups on the Common Metabolic Disease Knowledge Portal were adjusted for in two-step cis-MR, a method developed to address potential pleiotropy and linkage disequilibrium confounding. The method relies on splitting the MR estimate into an estimate contributed from the variant through the exposure, and an estimate contributed from the variant through the confounder, the latter is subtracted from the former to leave a theoretically unbiased overall estimate [27].

**Supplementary Figures**

**Figure 1. Association between genetically proxied PDE5 inhibition weighted by systolic blood pressure and magnetic resonance imaging brain traits.**


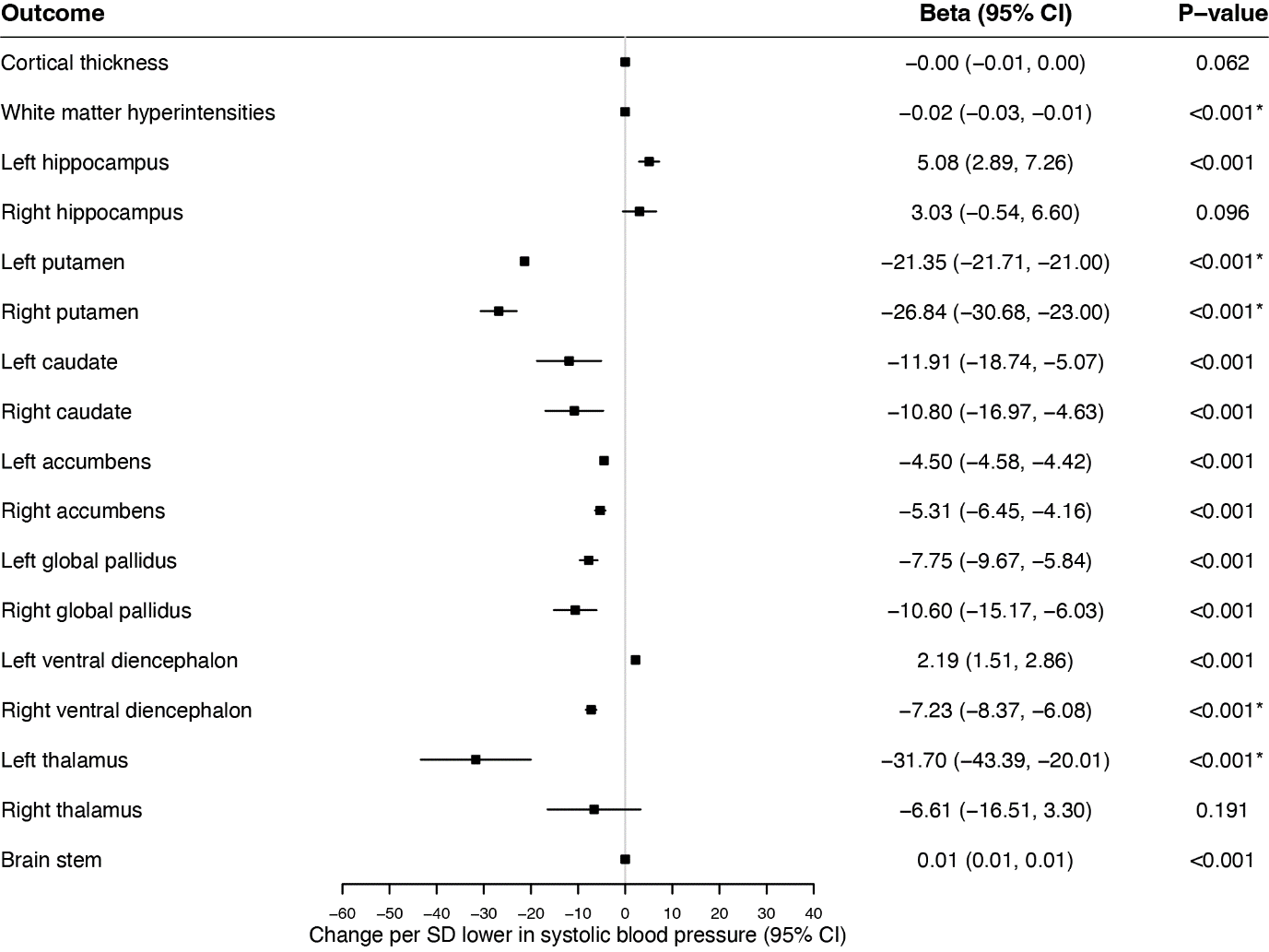


Results are derived from random-effects inverse-variance weighted Mendelian randomization analyses. OR indicates odds ratio; CI, confidence interval; SD, standard deviation. Outcomes marked with * indicates those that reached the sequential Bonferroni significance threshold (*p* <0.00011).

**Figure 2. Association between genetically proxied PDE5 inhibition weighted by PDE5A levels and magnetic resonance imaging brain traits.**


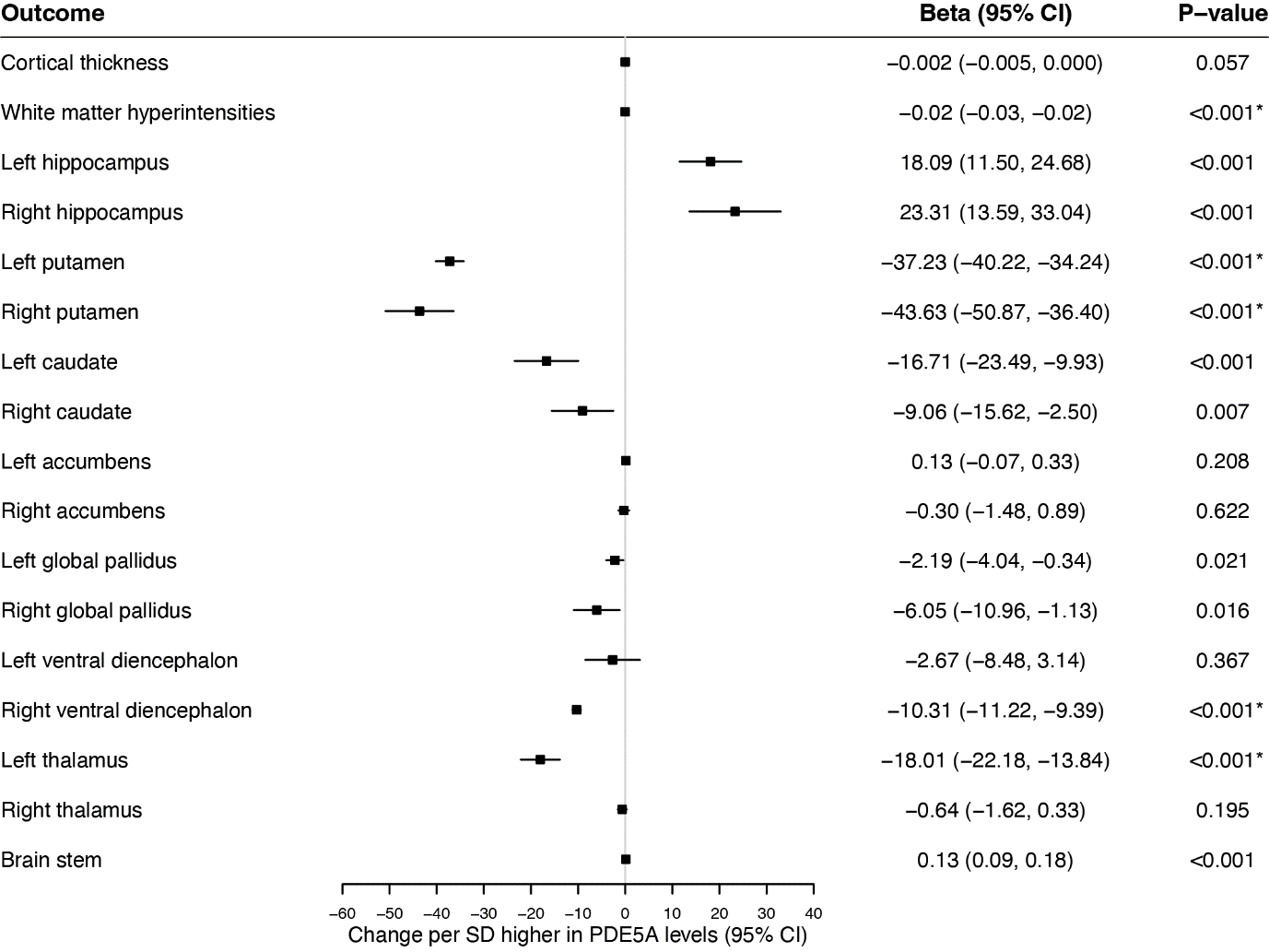


Results are derived from random-effects inverse-variance weighted Mendelian randomization analyses. OR indicates odds ratio; CI, confidence interval; SD, standard deviation. Outcomes marked with * indicates those that reached the sequential Bonferroni significance threshold (*p* <0.00011).

**Supplementary references**

[1] Lawlor DA, Harbord RM, Sterne JA, Timpson N, Davey Smith G (2008) Mendelian randomization: using genes as instruments for making causal inferences in epidemiology. Stat Med 27(8): 1133-1163. <https://doi.org/10.1002/sim.3034>

[2] Burgess S, Thompson SG (2011) Bias in causal estimates from Mendelian randomization studies with weak instruments. Stat Med 30(11): 1312-1323. <https://doi.org/10.1002/sim.4197>

[3] Gill D, Georgakis MK, Walker VM, et al. (2021) Mendelian randomization for studying the effects of perturbing drug targets. Wellcome Open Res 6: 16. <https://doi.org/10.12688/wellcomeopenres.16544.2>

[4] Burgess S, Davey Smith G, Davies NM, et al. (2019) Guidelines for performing Mendelian randomization investigations: update for summer 2023. Wellcome Open Res 4: 186. <https://doi.org/10.12688/wellcomeopenres.15555.3>

[5] Gill D, Walker VM, Martin RM, Davies NM, Tzoulaki I (2020) Comparison with randomized controlled trials as a strategy for evaluating instruments in Mendelian randomization. Int J Epidemiol 49(4): 1404-1406. <https://doi.org/10.1093/ije/dyz236>

[6] Evangelou E, Warren HR, Mosen-Ansorena D, et al. (2018) Genetic analysis of over 1 million people identifies 535 new loci associated with blood pressure traits. Nat Genet 50(10): 1412-1425. <https://doi.org/10.1038/s41588-018-0205-x>

[7] Sun BB, Chiou J, Traylor M, et al. (2023) Plasma proteomic associations with genetics and health in the UK Biobank. Nature 622(7982): 329-338. <https://doi.org/10.1038/s41586-023-06592-6>

[8] Bovijn J, Jackson L, Censin J, et al. (2019) GWAS Identifies Risk Locus for Erectile Dysfunction and Implicates Hypothalamic Neurobiology and Diabetes in Etiology. Am J Hum Genet 104(1): 157-163. <https://doi.org/10.1016/j.ajhg.2018.11.004>

[9] Galie N, Humbert M, Vachiery JL, et al. (2015) 2015 ESC/ERS Guidelines for the diagnosis and treatment of pulmonary hypertension: The Joint Task Force for the Diagnosis and Treatment of Pulmonary Hypertension of the European Society of Cardiology (ESC) and the European Respiratory Society (ERS): Endorsed by: Association for European Paediatric and Congenital Cardiology (AEPC), International Society for Heart and Lung Transplantation (ISHLT). Eur Respir J 46(4): 903-975. <https://doi.org/10.1183/13993003.01032-2015>

[10] Rhodes CJ, Batai K, Bleda M, et al. (2019) Genetic determinants of risk in pulmonary arterial hypertension: international genome-wide association studies and meta-analysis. Lancet Respir Med 7(3): 227-238. <https://doi.org/10.1016/S2213-2600(18)30409-0>

[11] Wightman DP, Jansen IE, Savage JE, et al. (2021) A genome-wide association study with 1,126,563 individuals identifies new risk loci for Alzheimer's disease. Nat Genet 53(9): 1276-1282. <https://doi.org/10.1038/s41588-021-00921-z>

[12] Kurki MI, Karjalainen J, Palta P, et al. (2023) FinnGen provides genetic insights from a well-phenotyped isolated population. Nature 613(7944): 508-518. <https://doi.org/10.1038/s41586-022-05473-8>

[13] Chia R, Sabir MS, Bandres-Ciga S, et al. (2021) Genome sequencing analysis identifies new loci associated with Lewy body dementia and provides insights into its genetic architecture. Nat Genet 53(3): 294-303. <https://doi.org/10.1038/s41588-021-00785-3>

[14] Grasby KL, Jahanshad N, Painter JN, et al. (2020) The genetic architecture of the human cerebral cortex. Science 367(6484). <https://doi.org/10.1126/science.aay6690>

[15] Smith SM, Douaud G, Chen W, et al. (2021) An expanded set of genome-wide association studies of brain imaging phenotypes in UK Biobank. Nat Neurosci 24(5): 737-745. <https://doi.org/10.1038/s41593-021-00826-4>

[16] Adamson CL, Alexander B, Ball G, et al. (2020) Parcellation of the neonatal cortex using Surface-based Melbourne Children's Regional Infant Brain atlases (M-CRIB-S). Sci Rep 10(1): 4359. <https://doi.org/10.1038/s41598-020-61326-2>

[17] Furtjes AE, Arathimos R, Coleman JRI, et al. (2023) General dimensions of human brain morphometry inferred from genome-wide association data. Hum Brain Mapp 44(8): 3311-3323. <https://doi.org/10.1002/hbm.26283>

[18] Borghammer P, Okkels N, Weintraub D (2024) Parkinson's Disease and Dementia with Lewy Bodies: One and the Same. J Parkinsons Dis 14(3): 383-397. <https://doi.org/10.3233/JPD-240002>

[19] Belbasis L, Morris S, van Duijn C, Bennett D, Walters R (2024) A Mendelian randomization study identifies proteins involved in neurodegenerative diseases. medRxiv: 2024.2002.2024.24303314. <https://doi.org/10.1101/2024.02.24.24303314>

[20] Elsworth B, Lyon M, Alexander T, et al. (2020) The MRC IEU OpenGWAS data infrastructure. bioRxiv: 2020.2008.2010.244293. <https://doi.org/10.1101/2020.08.10.244293>

[21] Vuckovic D, Bao EL, Akbari P, et al. (2020) The Polygenic and Monogenic Basis of Blood Traits and Diseases. Cell 182(5): 1214-1231 e1211. <https://doi.org/10.1016/j.cell.2020.08.008>

[22] van der Harst P, Verweij N (2018) Identification of 64 Novel Genetic Loci Provides an Expanded View on the Genetic Architecture of Coronary Artery Disease. Circ Res 122(3): 433-443. <https://doi.org/10.1161/CIRCRESAHA.117.312086>

[23] Chen MH, Raffield LM, Mousas A, et al. (2020) Trans-ethnic and Ancestry-Specific Blood-Cell Genetics in 746,667 Individuals from 5 Global Populations. Cell 182(5): 1198-1213 e1114. <https://doi.org/10.1016/j.cell.2020.06.045>

[24] Hemani G, Zheng J, Elsworth B, et al. (2018) The MR-Base platform supports systematic causal inference across the human phenome. Elife 7. <https://doi.org/10.7554/eLife.34408>

[25] Neale B (2018) Neale lab: UK Biobank. Available from <https://www.nealelab.is/uk-biobank/>. Accessed October 04 2024

[26] Bowden J, Del Greco MF, Minelli C, et al. (2019) Improving the accuracy of two-sample summary-data Mendelian randomization: moving beyond the NOME assumption. Int J Epidemiol 48(3): 728-742. <https://doi.org/10.1093/ije/dyy258>

[27] Woolf B, Zagkos L, Gill D (2022) TwoStepCisMR: A Novel Method and R Package for Attenuating Bias in cis-Mendelian Randomization Analyses. Genes (Basel) 13(9). <https://doi.org/10.3390/genes13091541>

**STROBE-MR checklist of recommended items to address in reports of Mendelian randomization studies**^1^ ^2^

| **Item No.** | **Section** | **Checklist item** | **Page No.** | **Relevant text from manuscript** |
| --- | --- | --- | --- | --- |
| 1 | **TITLE and ABSTRACT** | Indicate Mendelian randomization (MR) as the study’s design in the title and/or the abstract if that is a main purpose of the study | 1-2 | Inserted title and abstract |
|  | **INTRODUCTION** |  |  |  |
| 2 | **Background** | Explain the scientific background and rationale for the reported study. What is the exposure? Is a potential causal relationship between exposure and outcome plausible? Justify why MR is a helpful method to address the study question | 3-4 | Introduction |
| 3 | **Objectives** | State specific objectives clearly, including pre-specified causal hypotheses (if any). State that MR is a method that, under specific assumptions, intends to estimate causal effects | 3-4 | Introduction – final paragraph |
|  | **METHODS** |  |  |  |
| 4 | **Study design and data sources** | Present key elements of the study design early in the article. Consider including a table listing sources of data for all phases of the study. For each data source contributing to the analysis, describe the following: | 4 | Study design |
|  | a) | Setting: Describe the study design and the underlying population, if possible. Describe the setting, locations, and relevant dates, including periods of recruitment, exposure, follow-up, and data collection, when available. | 4 | Study design |
|  | b) | Participants: Give the eligibility criteria, and the sources and methods of selection of participants. Report the sample size, and whether any power or sample size calculations were carried out prior to the main analysis | 5-6 | Genetic instruments selection/Outcome data sources |
|  | c) | Describe measurement, quality control and selection of genetic variants | 5 | Genetic instruments selection |
|  | d) | For each exposure, outcome, and other relevant variables, describe methods of assessment and diagnostic criteria for diseases | 5/6 | Genetic instruments selection/Outcome data sources, Supplementary section: Data sources |
|  | e) | Provide details of ethics committee approval and participant informed consent, if relevant | 8 | Standard protocol approvals, registrations, and patient consents |
| 5 | **Assumptions** | Explicitly state the three core IV assumptions for the main analysis (relevance, independence and exclusion restriction) as well assumptions for any additional or sensitivity analysis | 7 | Statistical analysis/Sensitivity analysis |
| 6 | **Statistical methods: main analysis** | Describe statistical methods and statistics used | 7/8 | Statistical analysis/Sensitivity analysis/Reporting and packages |
|  | a) | Describe how quantitative variables were handled in the analyses (i.e., scale, units, model) | 7/8 | Reporting and packages |
|  | b) | Describe how genetic variants were handled in the analyses and, if applicable, how their weights were selected | 7 | Statistical analysis |
|  | c) | Describe the MR estimator (e.g. two-stage least squares, Wald ratio) and related statistics. Detail the included covariates and, in case of two-sample MR, whether the same covariate set was used for adjustment in the two samples | 7 | Statistical analysis |
|  | d) | Explain how missing data were addressed | NA |  |
|  | e) | If applicable, indicate how multiple testing was addressed | 8 | Reporting and packages |
| 7 | **Assessment of assumptions** | Describe any methods or prior knowledge used to assess the assumptions or justify their validity | 7 | Sensitivity analysis |
| 8 | **Sensitivity analyses and additional analyses** | Describe any sensitivity analyses or additional analyses performed (e.g. comparison of effect estimates from different approaches, independent replication, bias analytic techniques, validation of instruments, simulations) | 7 | Sensitivity analysis |
| 9 | **Software and pre-registration** |  |  |  |
|  | a) | Name statistical software and package(s), including version and settings used | 8 | Reporting and packages |
|  | b) | State whether the study protocol and details were pre-registered (as well as when and where) | NA |  |
|  | **RESULTS** |  |  |  |
| 10 | **Descriptive data** |  |  |  |
|  | a) | Report the numbers of individuals at each stage of included studies and reasons for exclusion. Consider use of a flow diagram | Figure 2 |  |
|  | b) | Report summary statistics for phenotypic exposure(s), outcome(s), and other relevant variables (e.g. means, SDs, proportions) | Supplementary  Tables | Supplementary Table 1, 2 |
|  | c) | If the data sources include meta-analyses of previous studies, provide the assessments of heterogeneity across these studies | NA |  |
|  | d) | For two-sample MR:  i.  Provide justification of the similarity of the genetic variant-exposure associations between the exposure and outcome samples  ii.  Provide information on the number of individuals who overlap between the exposure and outcome studies | NA  7/8  Supplementary Methods | Genetic instruments selection/Outcome data sources, Supplementary section: Data sources |
| 11 | **Main results** |  |  |  |
|  | a) | Report the associations between genetic variant and exposure, and between genetic variant and outcome, preferably on an interpretable scale | 9-11, Supplementary Tables | Results, Supplementary Table 1, 2 |
|  | b) | Report MR estimates of the relationship between exposure and outcome, and the measures of uncertainty from the MR analysis, on an interpretable scale, such as odds ratio or relative risk per SD difference | 9-11, Supplementary  Tables | Results, Supplementary Table 3-4, 6 |
|  | c) | If relevant, consider translating estimates of relative risk into absolute risk for a meaningful time period | NA |  |
|  | d) | Consider plots to visualize results (e.g. forest plot, scatterplot of associations between genetic variants and outcome versus between genetic variants and exposure) | Figure 2, 3, 4 |  |
| 12 | **Assessment of assumptions** |  |  |  |
|  | a) | Report the assessment of the validity of the assumptions | 9-11 | Results |
|  | b) | Report any additional statistics (e.g., assessments of heterogeneity across genetic variants, such as *I^2^*, Q statistic or E-value) | 9-11, Supplementary Tables | Results, Supplementary Table 3-4, 6 |
| 13 | **Sensitivity analyses and additional analyses** |  |  |  |
|  | a) | Report any sensitivity analyses to assess the robustness of the main results to violations of the assumptions | 10-11 | Results |
|  | b) | Report results from other sensitivity analyses or additional analyses | 11 | Results |
|  | c) | Report any assessment of direction of causal relationship (e.g., bidirectional MR) | 10 | Results |
|  | d) | When relevant, report and compare with estimates from non-MR analyses | NA |  |
|  | e) | Consider additional plots to visualize results (e.g., leave-one-out analyses) | Supplementary Tables | Supplementary Table 3-6 |
|  | **DISCUSSION** |  |  |  |
| 14 | **Key results** | Summarize key results with reference to study objectives | 12-16 | Discussion |
| 15 | **Limitations** | Discuss limitations of the study, taking into account the validity of the IV assumptions, other sources of potential bias, and imprecision. Discuss both direction and magnitude of any potential bias and any efforts to address them | 17 | Limitations |
| 16 | **Interpretation** |  |  |  |
|  | a) | Meaning: Give a cautious overall interpretation of results in the context of their limitations and in comparison with other studies | 12-15 | Discussion |
|  | b) | Mechanism: Discuss underlying biological mechanisms that could drive a potential causal relationship between the investigated exposure and the outcome, and whether the gene-environment equivalence assumption is reasonable. Use causal language carefully, clarifying that IV estimates may provide causal effects only under certain assumptions | 12-16 | Discussion |
|  | c) | Clinical relevance: Discuss whether the results have clinical or public policy relevance, and to what extent they inform effect sizes of possible interventions | 15-16 | Discussion |
| 17 | **Generalizability** | Discuss the generalizability of the study results (a) to other populations, (b) across other exposure periods/timings, and (c) across other levels of exposure | 16, 17 | Discussion, limitations |
|  | **OTHER INFORMATION** |  |  |  |
| 18 | **Funding** | Describe sources of funding and the role of funders in the present study and, if applicable, sources of funding for the databases and original study or studies on which the present study is based | 19 |  |
| 19 | **Data and data sharing** | Provide the data used to perform all analyses or report where and how the data can be accessed, and reference these sources in the article. Provide the statistical code needed to reproduce the results in the article, or report whether the code is publicly accessible and if so, where | 18 |  |
| 20 | **Conflicts of Interest** | All authors should declare all potential conflicts of interest | 18 |  |

This checklist is copyrighted by the Equator Network under the Creative Commons Attribution 3.0 Unported (CC BY 3.0) license.

1. Skrivankova VW, Richmond RC, Woolf BAR, Yarmolinsky J, Davies NM, Swanson SA, et al. Strengthening the Reporting of Observational Studies in Epidemiology using Mendelian Randomization (STROBE-MR) Statement. JAMA. 2021;under review.

2. Skrivankova VW, Richmond RC, Woolf BAR, Davies NM, Swanson SA, VanderWeele TJ, et al. Strengthening the Reporting of Observational Studies in Epidemiology using Mendelian Randomisation (STROBE-MR): Explanation and Elaboration. BMJ. 2021;375:n2233.
